# Dating of Pregnancy in First versus Second Trimester in Relation to Post-Term Birth Rate: A Cohort Study

**DOI:** 10.1371/journal.pone.0147109

**Published:** 2016-01-13

**Authors:** Ida Näslund Thagaard, Lone Krebs, Ulrik Lausten-Thomsen, Severin Olesen Larsen, Jens-Christian Holm, Michael Christiansen, Torben Larsen

**Affiliations:** 1 Department of Obstetrics and Gynaecology, Copenhagen University Hospital, Holbæk, Denmark; 2 Department of Clinical Biochemistry, State Serum Institute, Copenhagen, Denmark; 3 Department of Paediatrics, The Children’s Obesity Clinic, Copenhagen University Hospital, Holbæk, Denmark; University of Helsinki, FINLAND

## Abstract

**Objectives:**

To evaluate in a national standardised setting whether the performance of ultrasound dating during the first rather than the second trimester of pregnancy had consequences regarding the definition of pre- and post-term birth rates.

**Methods:**

A cohort study of 8,551 singleton pregnancies with spontaneous delivery was performed from 2006 to 2012 at Copenhagen University Hospital, Holbæk, Denmark. We determined the duration of pregnancy calculated by last menstrual period, crown rump length (CRL), biparietal diameter (1^st^ trimester), BPD (2^nd^ trimester), and head circumference and compared mean and median durations, the mean differences, the systematic discrepancies, and the percentages of pre-term and post-term pregnancies in relation to each method. The primary outcomes were post-term and pre-term birth rates defined by different dating methods.

**Results:**

The change from use of second to first trimester measurements for dating was associated with a significant increase in the rate of post-term deliveries from 2.1–2.9% and a significant decrease in the rate of pre-term deliveries from 5.4–4.6% caused by systematic discrepancies. Thereby 25.1% would pass 41 weeks when GA is defined by CRL and 17.3% when BPD (2^nd^ trimester) is used. Calibration for these discrepancies resulted in a lower post-term birth rate, from 3.1–1.4%, when first compared to second trimester dating was used.

**Conclusions:**

Systematic discrepancies were identified when biometric formulas were used to determine duration of pregnancy. This should be corrected in clinical practice to avoid an overestimation of post-term birth and unnecessary inductions when first trimester formulas are used.

## Introduction

The accurate dating of the duration of pregnancy is of importance in regard to prenatal care since several clinical decisions are based on gestational age (GA). In most industrialised countries, ultrasound (US) is accepted as the method of choice, yet in the literature, 7- to 14-day discrepancies are described. The discrepancy includes biological variation in the duration of pregnancy and methodological errors. [1, 2] Such a discrepancy has the potential to influence clinical decisions like the use of antenatal corticosteroid therapy early in pregnancy and labour induction in prolonged pregnancy.

US dating has been investigated in several studies, and biparietal diameter (BPD) in the second trimester has been found to be superior to last menstrual period (LMP). [1, 3, 4] The general use of crown rump length (CRL) during the first trimester is practiced in programmes in which first trimester risk calculations for Down’s syndrome are determined. CRL seems to be a reliable method, with fewer random and systematic errors compared to BPD. [2] Worldwide, there is a broad spectrum of formulas used for the calculation of GA, but no general consensus regarding which formula should be used.

In Denmark, an effort to standardise pregnancy dating has been made through the establishment of a national guideline. All pregnant women are offered a first trimester US scan, in which CRL and nuchal translucency (NT) are measured and GA is determined. This examination is a part of a free-of-charge prenatal programme combining age, biochemistry, and US screening for chromosomal abnormalities in the first trimester and a second trimester screening for malformations. The compliance rate of these two screenings has increased rapidly over the past few years, to the current 94%. [5] In 2007, the Danish Fetal Medicine Society decided to change the dating method from BPD measurement in the second trimester to CRL measurement in the first trimester. However, this change has not yet been properly evaluated. Previous studies of the use of BPD instead of LMP for dating show a reduced number defined as post-term pregnancies and thus a reduction of induction rates. [4, 6, 7]

The aim of the present study was to compare dating during the first and second trimester and determine their consequences for rates of pre-term and post-term births.

## Material and Methods

The results of this cohort study were reported following the STROBE recommendation. The study was approved by the Danish Data Protection Agency and the regional Research Ethics board (reg. no. SJ-HO-01 and SJ-335). Written informed consent was not required and therefore not obtained. However, patients record and the data were anonymized before analysis.

Data were collected prospectively from 2006 to 2012 at Copenhagen University Hospital, Holbæk, in Denmark. The population was an unselected population of pregnant women, and data were retrieved from the Astraia database (www.astraia.com), which comprised all US examinations performed on pregnant women during the study period and includes the mothers’ medical history, LMP, parity, pre-pregnancy maternal weight and height, and smoking and alcohol habits. The data were linked by the personal civil registration number with data from the Danish Medical Birth Registry, which contains data on the date of birth, birth weight and length, infant sex, number of infants born, intrauterine foetal death, pregnancy loss and complications during birth. All births in Denmark are registered in the Danish Medical Birth Registry and at the time of data extraction the register were administered by the Danish National Board of Health. [8]

**Fig 1:**
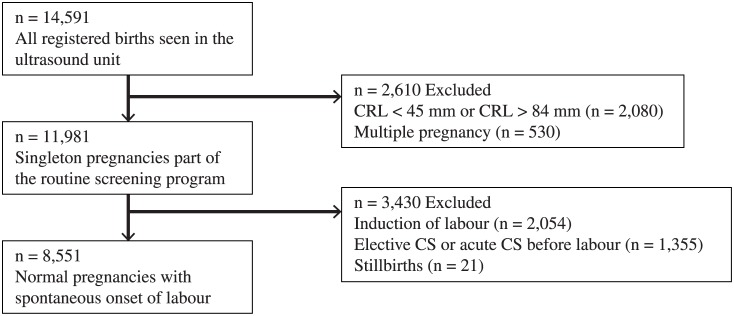
Included and excluded patients in the study.

During the study period 14,591 women visited our US unit and subsequently gave birth to a child. We identified all pregnancies in which the CRL was measured to be between 45–84 mm, which is the interval used as the standard criterion for pregnancy dating according to the Fetal Medicine Foundation criterion. (https://fetalmedicine.org/nuchal-translucency-scan) Inclusion criteria were a registered CRL and the later birth of a live-born child. The following pregnancies were excluded: a CRL registered < 45 mm and > 84 mm (*n* = 2,080), multiple pregnancies (*n* = 530), induction of labour (*n* = 2,054), elective caesarean section (CS) or acute CS before labour (*n* = 1,355), and stillbirths (*n* = 21). A total of 8,551 pregnancies were included in the study. (Fig 1)

BPD and HC measurements were categorized in the following intervals: BPD_1_ between 19–30 mm (GA: 11–14 + 6), BPD_2_ between 31–55 mm (GA: 15–22), and HC 110–200 mm (GA: 15–22). Sub-analyses were performed in which unregistered LMP (487), BPD_1_ (132), BPD_2_ (367), and HC (822) were excluded when analyses included one of these parameters. Absence of this information meant that foetometry was not performed during the given interval, or that one of the parameters was not registered.

The GA was calculated using the 1^st^ day of the LMP, the CRL (Robinson and Fleming formula) [9], and the BPD and HC (formulas of Chitty et al.). [10]

These formulas were chosen since they are in accordance with the Danish national guidelines. GA at birth was computed from GA defined at the time of the US examination (depending on method used) plus the number of days from the US scan to the date of birth.

The biometric measurements were done in accordance with the instructions provided by the authors of the above-mentioned formulas. The ultrasound examinations were all performed by midwives and doctors trained and certificated to perform NT measurements by the Fetal Medical Foundation, London, UK.

A delivery at ≤ 258 completed days was considered pre-term, a delivery between 259–293 completed days to be term, and a delivery at ≥ 294 completed days to be post-term.

CRL and BPD_1_ in the first and BPD_2_ and HC in the second trimesters were analysed by comparing GAs (mean, median, mode, and standard deviation (SD)) at spontaneous delivery, defined by the date of birth and a spontaneous onset of birth. The methods were tested for mean differences compared to different methods to take account for the normal variation of the pregnancy length. The mean difference is the methodological differences and SD is the random insecurity in-between the methods.

To evaluate systematic errors in the prediction models, the errors were calculated by subtracting the predicted date from the median duration of the pregnancy as suggested by Okland *et al*. [11] The median pregnancy length was set to 280 days. The formulas were then calibrated by subtracting the systematic discrepancies from the estimated GA. A sensitivity analyse was performed including the induced pregnancies. This did not affect the median pregnancy length and thereby the systematic discrepancies (data not shown). The induction rates were also calculated (S1 Table).

The percentages of deliveries with GAs of ≤ 247, ≤ 258, ≥ 287, ≥ 290, and ≥ 294 on the day of birth were compared between the methods CRL, BPD_1_, BPD_2_, HC, and LMP.

McNemar’s test was used in analysing paired data. All statistical analyses were performed using SPSS, version 21. All tests were two-tailed and a *p* value of < 0.05 was considered statistically significant.

## Results

There were up to three days differences in median gestational age depending on the method employed. The SD was smallest for first trimester dating (Table 1).

**Table 1:** Mean, median, and mode of Pregnancy length (days) in relation to dating method.

Variable	Number of pregnancies (%)	Mean	Median	Mode	SD
**LMP**	**8064 (94.3%)**	**280.7**	**282**	**284**	**13.9**
**CRL**	**8551 (100%)**	**279.3**	**281**	**285**	**12.3**
**BPD**_**1**_	**8419 (98.4%)**	**278.9**	**281**	**284**	**12.3**
**BPD**_**2**_	**8309 (97.2%)**	**277.1**	**279**	**281**	**12.6**
**HC**	**7729 (90.4%)**	**276.6**	**279**	**280**	**12.5**

BPD_1_ measurement in 1st trimester, BPD_2_ measurement in 2nd trimester

Table 2 shows the mean difference between first trimester dating by CRL and second trimester dating by BPD. First trimester measurements were superior with the smallest mean difference and SD (0.38, SD 2,04).

**Table 2:** Measurement errors when methods were compared to first and second trimester measurements.

Mean differences of CRL compared with other methods					Mean differences of BPD_2_ compared with other methods				
Method	Mean difference	SD	SDE	p	Method	Mean difference	SD	SDE	p
**LMP**	**-1.45**	**6.94**	**0.08**	**< 0.001**	**LMP**	**-3.64**	**7.85**	**0.09**	**< 0.001**
**BPD**_**1**_	**0.38**	**2.04**	**0.02**	**< 0.001**	**BPD**_**1**_	**-1.78**	**3.91**	**0.04**	**< 0.001**
**BPD**_**2**_	**2.19**	**3.98**	**0.04**	**< 0.001**	**CRL**	**-2.19**	**3.98**	**0.04**	**< 0.001**
**HC**	**2.78**	**3.15**	**0.04**	**< 0.001**	**HC**	**0.61**	**2.38**	**0.03**	**< 0.001**

BPD_1_ measurement in 1st trimester, BPD_2_ measurement in 2nd trimester

The systematic discrepancies (defined as median difference by subtracting the medians in Table 1 from 280) were calculated to -2 days for LMP, -1 day for CRL and BPD_1_, and +1 day for BPD_2_ and HC.

**Fig 2:**
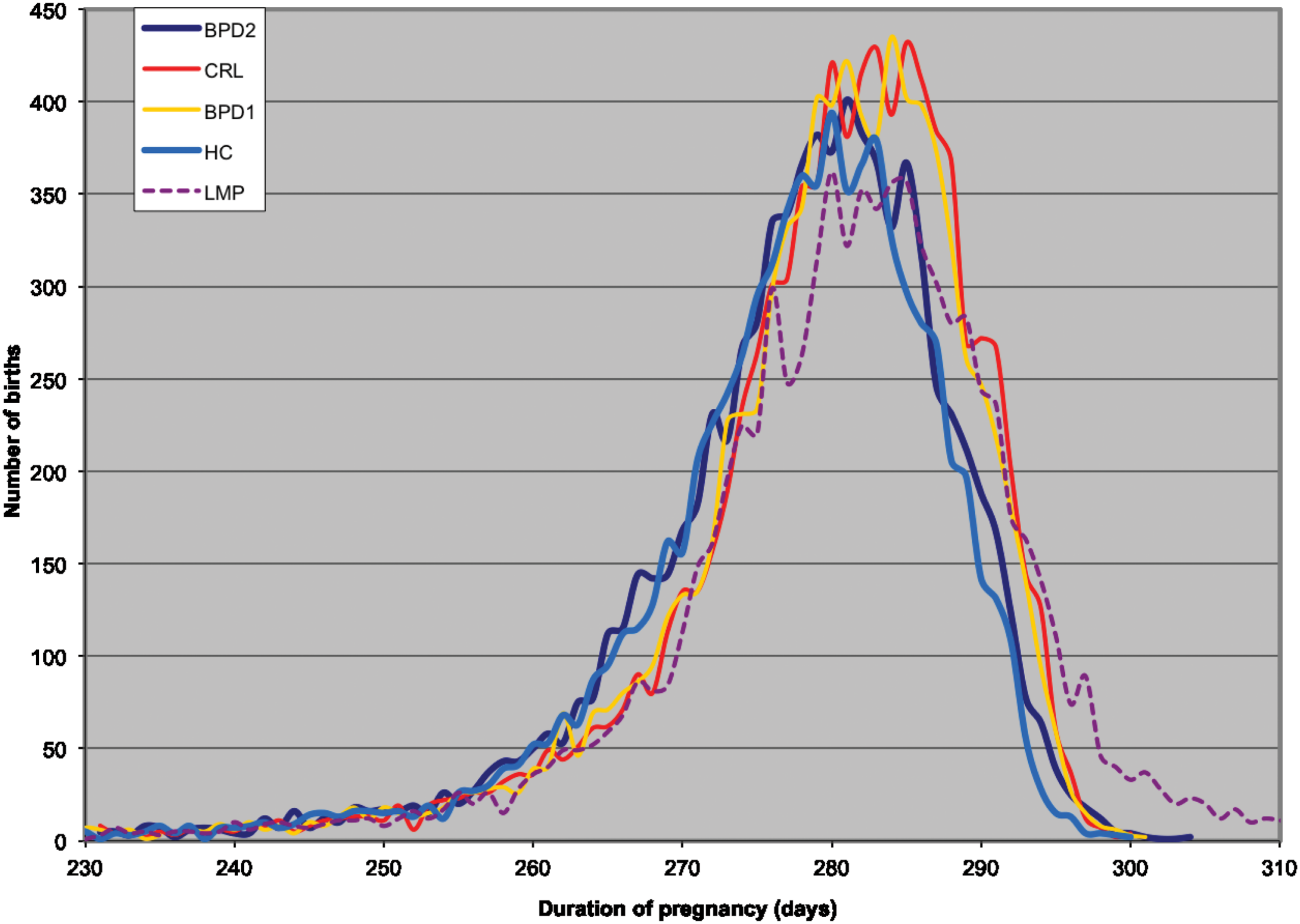
The distribution of gestational age at birth in relation to dating method.

Table 3 shows the distribution of pregnancies defined as pre-term, term, and post-term in relation to the five different methods tested. There was a left-side shift in the distribution of second trimester measurement compared to first trimester (Fig 2), indicating a significantly different number of pre- and post-term pregnancies. HC (2^nd^ trimester) defined most pregnancies within the interval of term (93.6%), BPD_1_, (92.8%), BPD_2_ (92.2%), CRL (92.2%) and LMP (86.0%). LMP defined most pregnancies as post-term (9.9%) and HC fewest (0.9%), both significantly different compared to CRL.

**Table 3:** Number of pre-term, term, and post-term pregnancies in relation to dating method.

		Prediction method													
Definition	GA at birth (days)	CRL		BPD_1_			BPD_2_			HC			LMP		
		N	%	n	%	*p* ^3^	n	%	*p* ^3^	n	%	*p* ^3^	n	%	*p* ^3^
**Pre-term**	**≤ 237**	**103**	**1.2**	**99**	**1.2**	**< 0.001**	**107**	**1.3**	**< 0.001**	**106**	**1.4**	**< 0.001**	**96**	**1.2**	**< 0.001**
**Pre-term**	**> 247 to ≤ 258**	**288**	**3.4**	**297**	**3.5**	**< 0.001**	**339**	**4.1**	**< 0.001**	**320**	**4.1**	**< 0.001**	**254**	**3.1**	**< 0.001**
**Term**	**> 258 to < 287**	**6006**	**70.0**	**6067**	**72.1**	**< 0.001**	**6321**	**77.2**	**< 0.001**	**6126**	**79.3**	**< 0.001**	**5232**	**64.9**	**< 0.001**
**Term**	**≥ 287 to < 290**	**1021**	**11.9**	**957**	**11.4**	**0.211**	**689**	**8.4**	**< 0.001**	**672**	**8.7**	**< 0.001**	**865**	**10.7**	**< 0.001**
**Term**	**≥ 290 to < 294**	**884**	**10.3**	**785**	**9.3**	**< 0.001**	**555**	**6.8**	**< 0.001**	**436**	**5.6**	**< 0.001**	**818**	**10.4**	**< 0.001**
**Post-term**	**≥ 294**	**249**	**2.9**	**214**	**2.5**	**0.205**	**173**	**2.1**	**< 0.001**	**69**	**0.9**	**< 0.001**	**799**	**9.9**	**< 0.001**
	**Total**	**8551**		**8419**			**8184**			**7729**			**8064**		
	**Missing**	**0**		**132**			**367**			**822**			**487**		

BPD_1_ measurement in 1st trimester, BPD_2_ measurement in 2nd trimester; ^3^ *p*-value when comparing differences with CRL

Calibration of the prediction models showed that the distributions of pregnancies defined as pre-term, term, and post-term were more even, especially between CRL and BPD_2_. However, there was still a significant difference in post-term rate between CRL and BPD_2_, with an increase in pregnancies defined as post-term by BPD_2_ (3.1%) (Table 4). The distributions of defining term pregnancies were similar for all US biometrics (CRL (93.5%), BPD_1_ (93.5%), BPD_2_ (92.0%), HC (93.4%)), but different for LMP (88.1%).

**Table 4:** Number of pre-term, term, and post-term pregnancies in relation to dating method after calibration of formulas. Each method is compared with CRL.

		Prediction method													
Definition	GA at birth (days)	CRL		BPD_1_			BPD_2_			HC			LMP		
		N	%	n	%	*p* ^3^	n	%	*p* ^3^	n	%	*p* ^3^	n	%	*p* ^3^
**Pre-term**	**≤ 237**	**107**	**1.3**	**104**	**1.2**	**0.724**	**103**	**1.2**	**0.839**	**101**	**1.3**	**0.361**	**105**	**1.3**	**0.839**
**Pre-term**	**> 237 to ≤ 258**	**320**	**3.7**	**318**	**3.8**	**0.892**	**304**	**3.7**	**0.934**	**292**	**3.8**	**0.019**	**309**	**3.8**	**0.239**
**Term**	**> 258 to < 287**	**6354**	**74.3**	**6414**	**76.2**	**< 0.001**	**6124**	**73.7**	**0.138**	**5920**	**76.1**	**< 0.001**	**5750**	**71.3**	**< 0.001**
**Term**	**≥ 287 to < 290**	**909**	**10.6**	**831**	**9.8**	**< 0.001**	**813**	**9.8**	**0.044**	**760**	**9.8**	**< 0.001**	**763**	**9.5**	**0.006**
**Term**	**≥ 290 to < 294**	**738**	**8.6**	**632**	**7.5**	**< 0.001**	**705**	**8.5**	**0.533**	**581**	**7.5**	**< 0.001**	**590**	**7.3**	**< 0.001**
**Post-term**	**≥ 294**	**123**	**1.4**	**120**	**1.4**	**1.000**	**260**	**3.1**	**< 0.001**	**125**	**1.6**	**0.938**	**547**	**6.8**	**< 0.001**
	**Total**	**8551**		**8419**			**8309**			**7779**			**8064**		
	**Missing**	**0**		**132**			**242**			**772**			**487**		

BPD_1_ measurement in 1st trimester, BPD_2_ measurement in 2nd trimester; ^3^ *p*-value when comparing differences with CRL

## Discussion

In a large cohort of pregnant women, the use of first trimester dating resulted in significantly more post-term pregnancies compared to second trimester dating.

This is of special interest due to the on-going discussion on whether induction of labour should be performed at 41 + 0 instead of 42 + 0 weeks in low risk pregnancies. We found a significant decrease in post-term birth rate when comparing LMP to BPD dating in the second trimester, which is in agreement with previous studies. [12] However, the same effect has not been described when comparing second trimester with first trimester US dating even though it has been hypothesised. [1, 13] In the present study, the post-term birth rate increased by using CRL (2.9%), compared to BPD_2_ (2.1%). Though, when the formulas in our material were calibrated to a duration of pregnancy of 280 days, the opposite was found. [11]

Our results showed that if the time of induction was changed from 42 + 0 to 41 + 0, the difference between the percentages of pregnancies reaching 41+ 0 defined by first versus second trimester calculation increased to 7.8%. This means that 25.1% of all pregnancies would pass 41 weeks when GA is defined by CRL, 17.3% when BPD_2_ is used, and 31% when LMP is used.

When calibrating the formulas by assuming that the median duration of pregnancy should be 280 days, the post-term birth rate decreased using first trimester biometrics. This is not standard in our national setting, but should be considered to diminish these differences. Alternatively, other and more precise formulas could be used as population-based prediction methods. [14, 15] The median duration of pregnancy used for calibration could be estimated to be 281 or 282. In the literature, there are several studies that consider this to be a better estimate. [3, 4, 13] In our study, first trimester measurements support a median length of 281 days, while LMP supports a length of 282 days.

The current literature on this issue is relatively sparse and other studies have suggested that dating in first trimester with CRL would decrease post-term birth rate further, but this association has not been substantiated. Taipale *et al*. argued that first trimester US would theoretically decrease post-term rates compared to second trimester US; however, the study did not include second trimester measurements. [1] In a randomised trial comparing CRL and LMP, no significant difference in rate of induction for prolonged pregnancy was found, but the study had problems with recruitment, including only 468 of the 800 expected. [16] Saltvendt *et al*. found a lower post-term birth rate in first trimester compared to second trimester measurement, though the difference was not significant. [13]

There is a need to establish standardised clinical guidelines for surveillance and intervention during pregnancy. Worldwide, there are different weeks for schedules of US, formulas, and methods used for dating. In the present study, first trimester measurements CRL and BPD_1_ showed identical results regarding the duration of pregnancy (median and SD) and a mean difference of 0.38 (SD: 2.04) days, saying first trimester dating is more precise and with the smallest variation. In our regimen, we measure BPD routinely in the first trimester in order to detect central nervous defects. Therefore it may be easier for inexperienced personnel to measure BPD instead of CRL.

The dating methods are based on the assumption that all foetuses are of similar size at a given GA during the first half of the pregnancy, yet several factors, e.g. gender, parity, maternal age, smoking habits, etc., may influence dating. [12, 17, 18] On the other hand, US has been proven to be a robust method with high reproducibility when performed by experienced personnel, with small intra-/inter observer error. [17] It is important to notice that US formulas used in the second trimester are not always representative for the first trimester. [13] Chitty *et al*. have published two formulas [10, 19], which we compared with our data. Like Saltvedt *et al*. we found that GAs were skewed to the right in both the first and second trimesters when using the second formula from 1997, giving a median in BPD_1_ of 284 and 285 in BPD_2_ (data not shown). [13, 19] However, after calibration, the two formulas performed similarly.

The strength of the present study is that we studied a large unselected population in a standardised programme with a large number of first trimester measurements, thereby minimising potential selection bias. Exclusion of post-term induction is a limitation to the study, causing an artificially low post-term rate. By using an older cohort this selection bias was minimized, since induction-rate in general is increasing. In our material the mean induction-rate was 17.1% (S1 Table) compared to an induction-rate in Denmark of 25% in 2014. Defining the pregnancy length by mean, median or mode is widely debated. Mean values are affected by preterm births and also inductions due to post-term pregnancies; median values are not affected and therefore considered the most representative value estimating the general pregnancy length. [13] Mode is sensible to the number of pregnancies and therfore narrowly used. [3] We evaluated both on mean and median pregnancy length and found the same systematic discrepancies regradles of the statistical approaches including adding induction to the median pregnancy length. We found a significant effect on change in post-term rate compared to different dating methods. If we have had a lower induction rate in Denmark we are confident that the effect would be larger but it would not change our conclusion.

In conclusion, we found an increase of the post-term rate in dating performed in first trimester compared to second trimester, caused by systematic discrepancies. When the formulas were calibrated, the post-term birth rates in the measurements performed in first trimester decreased. It is important to evaluate and correct the formulas used for dating to avoid misclassification of pre-term and post-term pregnancies and thus avoid unnecessary inductions due to post-term pregnancy.

## Supporting Information

S1 Table Number of inductions, caesarean section and spontaneous deliveries per year. (DOCX)
