# Supplementary material for: Dating of Pregnancy in First versus Second Trimester in Relation to Post-Term Birth Rate: A Cohort Study
Source: PLoS One. 2016 Jan 13;11(1):e0147109. doi: 10.1371/journal.pone.0147109 (PMC4711898; doi:10.1371/journal.pone.0147109)
Supplement: S1 Table — (DOCX) [file pone.0147109.s001.docx]

**S1 Table Number of inductions, caesarean section and spontaneous deliveries per year**

|  | **2006** | **2007** | **2008** | **2009** | **2010** | **2011** | **Total** |
| --- | --- | --- | --- | --- | --- | --- | --- |
| **Induction N (%)** | **126 (12.2)** | **314 (13.9)** | **378 (15.8)** | **436 (18.8)** | **393 (17.7)** | **407 (23.2)** | **2054 (17.1)** |
| **Spontaneus delivery N (%)** | **789 (76.4)** | **1708 (75.8)** | **1711 (72.7)** | **1609 (69.4)** | **1591 (72.8)** | **1143 (65.3)** | **8551 (71.5)** |
| **Caesarean section before labour N (%)** | **118 (11.4)** | **230 (10.2)** | **298 (12.5)** | **275 (11.9)** | **233 (10.5)** | **201 (11.5)** | **1355 (11.3)** |
| **Total** | **1033** | **2252** | **2387** | **2320** | **2217** | **1751** | **11960** |
